# Supplementary material for: Efficacy and Adverse Events in Metastatic Melanoma Patients Treated with Combination BRAF Plus MEK Inhibitors Versus BRAF Inhibitors: A Systematic Review
Source: Cancers (Basel). 2019 Dec 5;11(12):1950. doi: 10.3390/cancers11121950 (PMC6966686; doi:10.3390/cancers11121950)
Supplement: Supplementary file 1 [file cancers-11-01950-s001.pdf]

cancers

Supplementary Materials

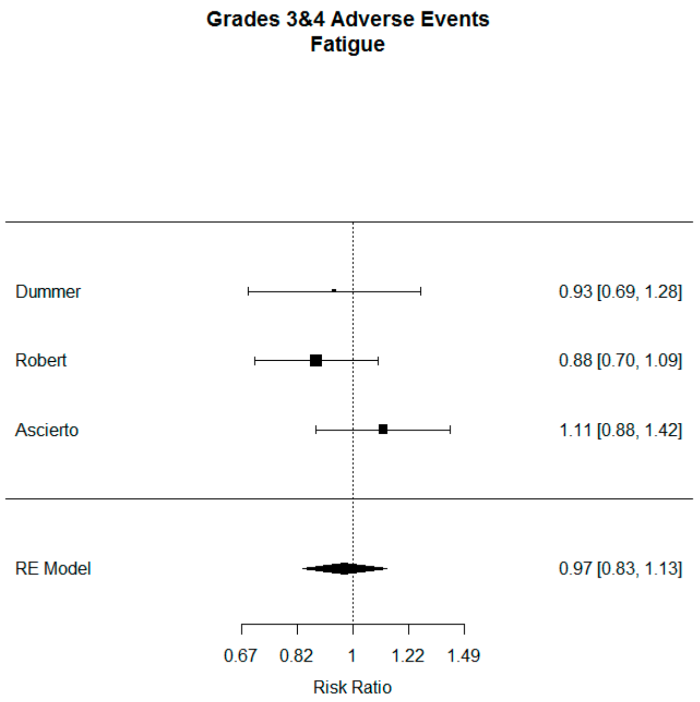

Figure S1. Relative risk (RR) of "fatigue" adverse events; grades 3 and 4. Combined RR of 0.97 (95% CI 0.83–1.13) showed no significant difference in risk of fatigue between combination and monotherapy. Relative risk in individual studies also showed no significant difference in risk of grade 3 and 4 adverse events of fatigue between groups.

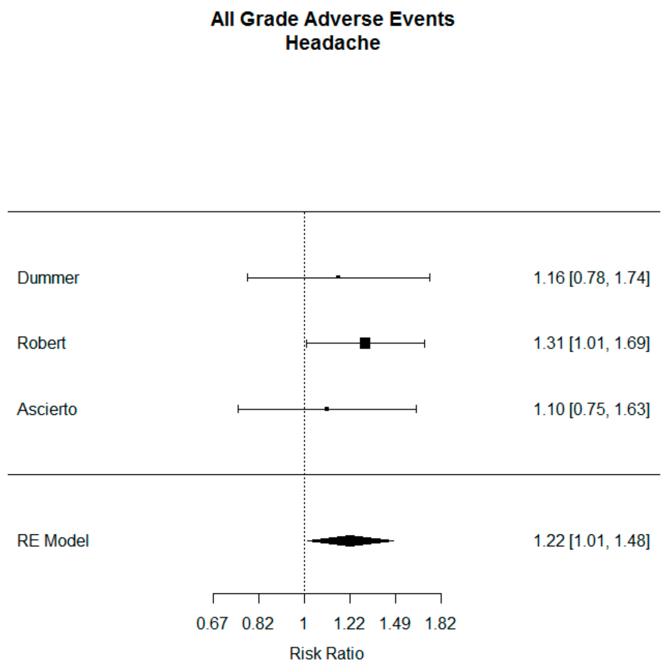

Figure S2. Relative risk (RR) of "headache" adverse events; all grades. Combined relative risk (RR) of 1.21 (95% CI 1.01–1.48) showed a significantly higher risk of developing an adverse event due to headache in the combination group. Dabrafenib plus trametinib in the Robert study had a RR of 1.31 (95% CI 1.01–1.69), revealing a significantly higher risk of headache in the combination group compared to vemurafenib. The other combination groups showed no significant difference in risk of headache when compared to vemurafenib.

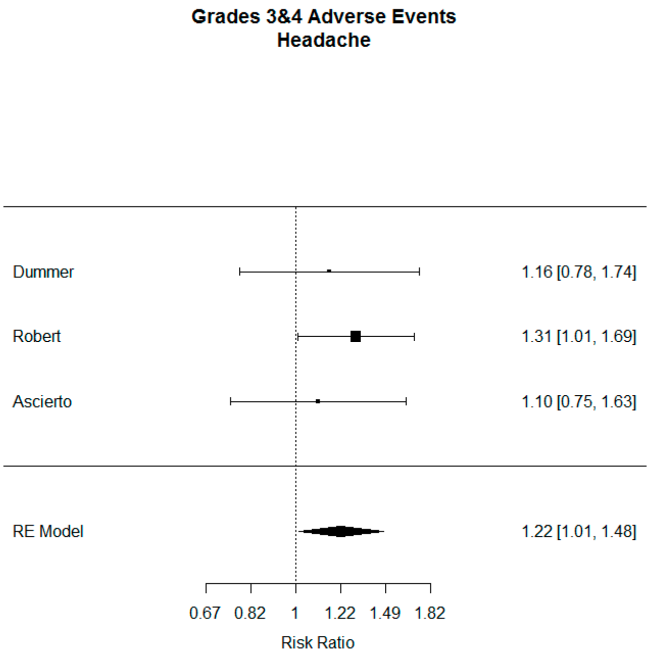

Figure S3. Relative risk (RR) of "headache" adverse events; grades 3 and 4. Combined RR of 1.21 (95% CI 1.01–1.48) showed a significantly higher risk of developing an adverse event due to headache in the combination group. Dabrafenib plus trametinib in the Robert study had a RR of 1.31 (95% CI 1.01–1.69), revealing a significantly higher risk of headache in the combination group compared to vemurafenib. The other combination groups showed no significant difference in risk of headache when compared to vemurafenib.

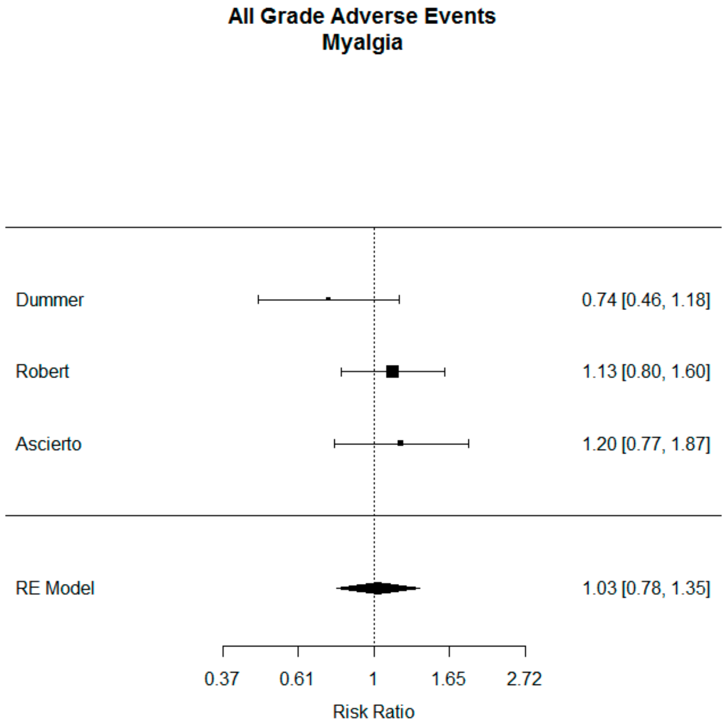

Figure S4. Relative risk (RR) of "myalgia" adverse events; all grades. Combined RR of 1.03 (95% CI 0.78–1.35) showed no significant difference in risk of fatigue between combination and monotherapy. Relative risk in individual studies also showed no significant difference in risk of myalgia between groups.

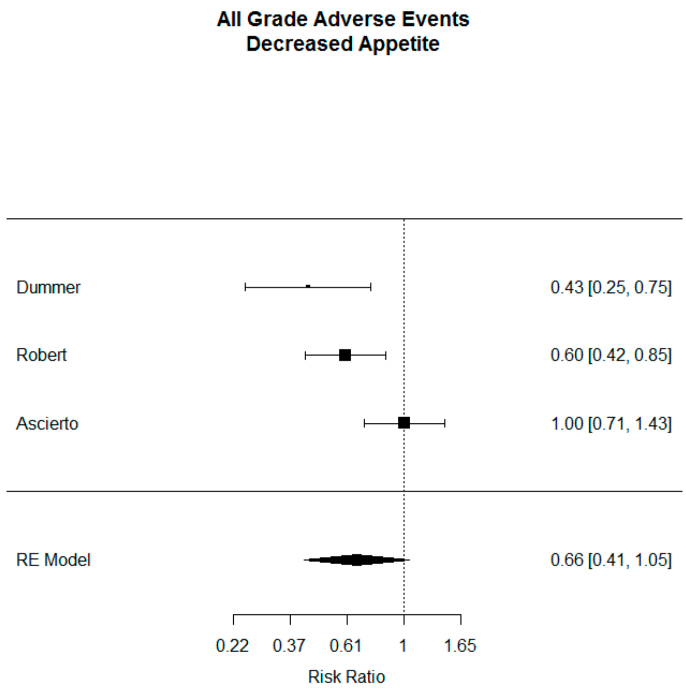

Figure S5. Relative risk (RR) of "decreased appetite" adverse events; all grades. Combined RR of 0.66 (95% CI 0.41–1.05) showed no significant difference in risk of decreased appetite between combination and monotherapy. Encorafenib plus binimetinib in the Dummer study had an RR of 0.43 (95% CI 0.25–0.75), revealing a significantly smaller risk of decreased appetite as compare to monotherapy. Dabrafenib plus trametinib in the Robert study had an RR of 0.60 (95% CI 0.42–0.85), revealing a significantly smaller risk of decreased appetite in the combination group. Vemurafenib and cobimetinib in the Ascierto study did not show any significant difference in risk of decreased appetite between groups.

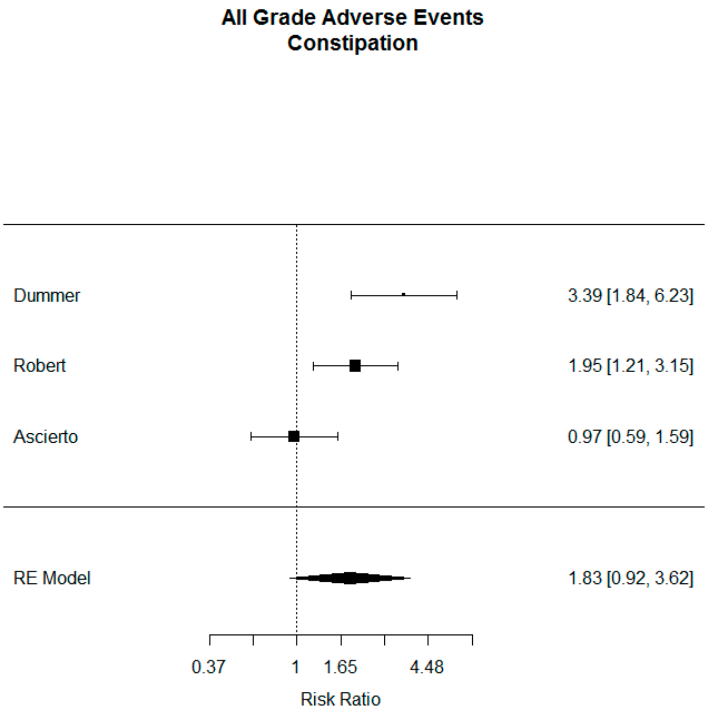

Figure S6. Relative risk (RR) of "constipation" adverse events; all grades. An RR of 1.83 (95% CI 0.9–3.62) was found overall in combination compared to monotherapy and was not statistically significant. In the Dummer (RR: 3.39, 95% CI 1.84–6.23) and Robert (RR: 1.95, 95% CI 1.21–3.15) trials, there was higher risk of constipation in the combined group over the monotherapy group, which was statistically significant. In the Ascierto trial (RR: 0.97, 95% CI 0.59–1.59), it was relatively equal in both

groups and was not statistically significant. It can also be said that encorafenib plus binimetinib had a significantly higher risk of constipation than the combination of vemurafenib plus cobimetinib, as confidence intervals for respective relative risks are not overlapping and are compared to a monotherapy-like group. Vemurafenib plus cobimetinib showed a significantly higher risk of nausea than the combination of dabrafenib plus trametinib.

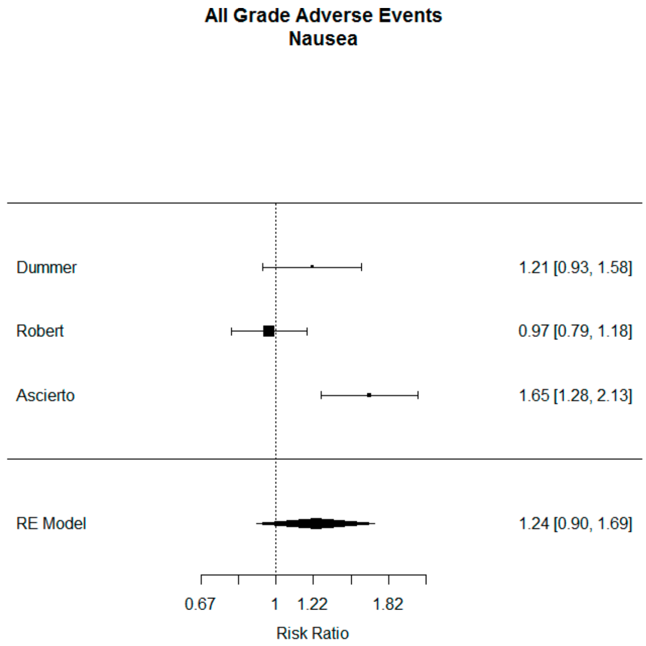

Figure S7. Relative risk (RR) of "nausea" adverse events; all grades. Nausea was relatively 1.24 (95% CI 0.90–1.69) times more common in combined therapy over monotherapy. There was no statistical significance in the Dummer (RR: 1.21, 95% CI 0.93–1.58) or Robert (RR: 0.97, 95% CI 0.79–1.18) trials. However, in the Ascierto trial, it was relatively 1.65 (95% CI 1.28–2.13) times more common in combined therapy over monotherapy, which was statistically significant. It can also be said that vemurafenib plus cobimetinib had a significantly higher risk of nausea than the combination of dabrafenib plus trametinib, as confidence intervals for respective relative risks are not overlapping and are compared to a like mono-therapy group.

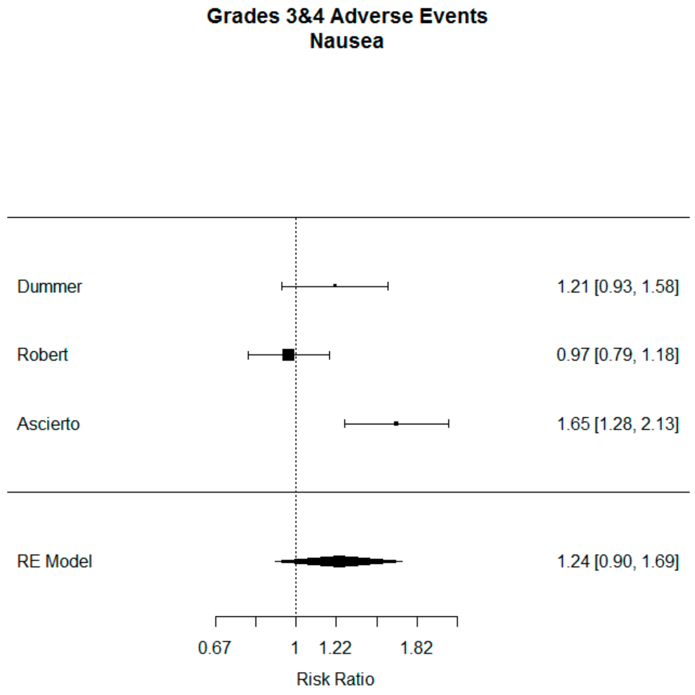

Figure S8. Relative risk (RR) of "nausea" adverse events; grades 3 and 4. Nausea was 1.24 (95% CI 0.90–1.69) times more common in combined therapy over monotherapy. RR in Dummer 1.21 (95% CI 0.93–1.58), Robert 0.97 (95% CI 0.79–1.18), and Ascierto 1.65 (95% CI 1.28–2.13). Only the Ascierto trial showed statistical significance. It can also be said that vemurafenib plus cobimetinib had a significantly higher risk of nausea than the combination of dabrafenib plus trametinib, as confidence intervals for respective relative risks are not overlapping and are compared to a monotherapy-like group.

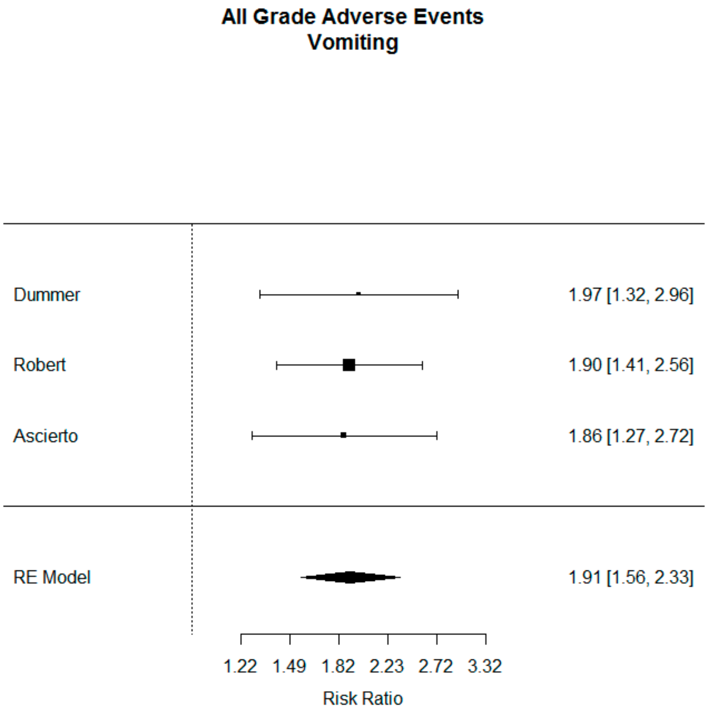

Figure S9. Relative risk (RR) of "vomiting" adverse events; all grades. Vomiting was relatively 1.91 (95% CI 1.56–2.33) times more common in combination over monotherapy and was statistically significant. Similar results are shown in all trials; Dummer (RR: 1.97, 95% CI 1.32–2.96), Robert (RR: 1.90, 95% CI 1.41–2.56), and Ascierto (RR: 1.86, 95% CI 1.27–2.72), showing vomiting more common in combined therapy over monotherapy.

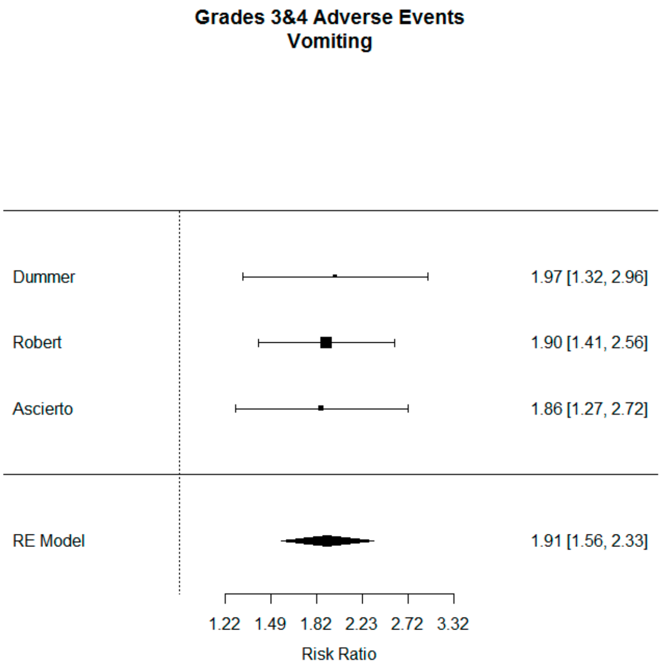

Figure S10. Relative risk (RR) of "vomiting" adverse events; grades 3 and 4. Vomiting was 1.91 (95% CI 1.56–2.33) times more common in combined therapy over monotherapy. All trials showed vomiting was more common in monotherapy and was statistically significant. RR in Dummer was 1.97 (95% CI 1.32–2.96), Robert 1.90 (95% CI 1.41–2.56), and Ascierto 1.86 (95% CI 1.27–2.72).

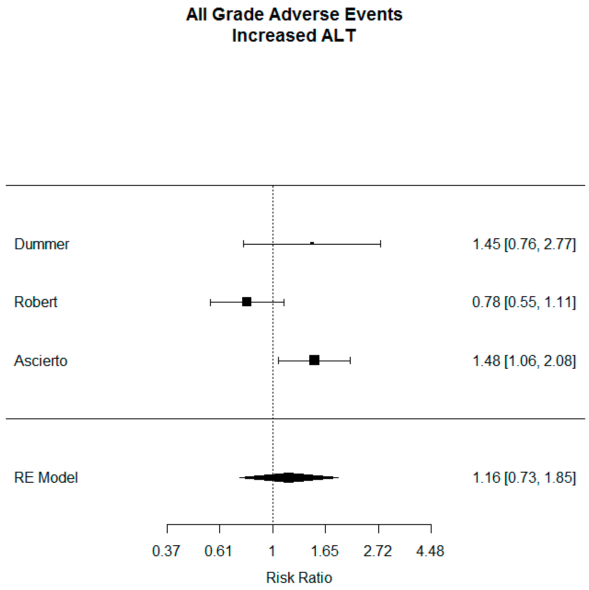

Figure S11. Relative risk (RR) of "increased ALT" adverse events; all grades. Increase ALT was relatively 1.16 (95% CI 0.73–1.85) times more common in combined therapy over monotherapy. Both the Dummer (RR: 1.45, 95% CI 0.76–2.77) and Robert (RR: 0.78, 95% CI 0.55–1.11) trials showed no statistical significance, but in the Dummer trial, it was more common in combined therapy over monotherapy. In the Ascierto trial, it was relatively 1.48 (1.06–2.08) times more common in combined therapy over monotherapy and was statistically significant.

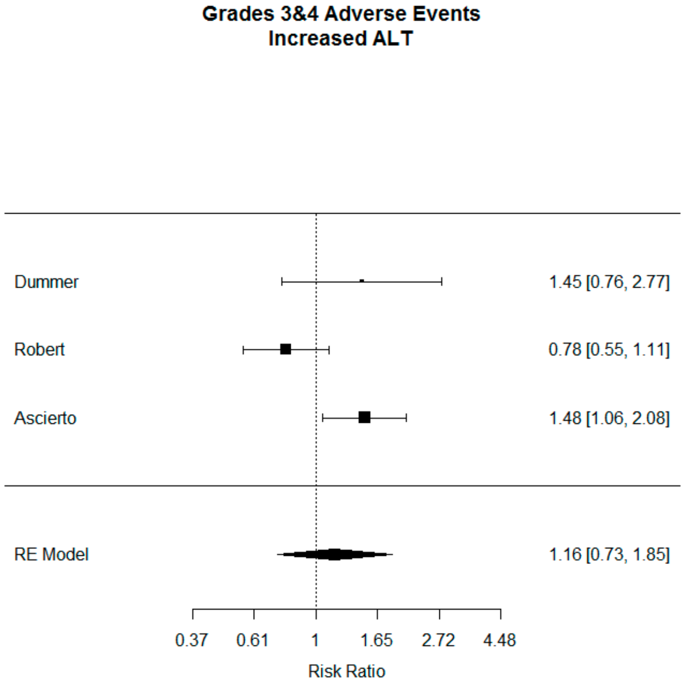

Figure S12. Relative risk (RR) of "increased ALT" adverse events; grades 3 and 4. Increased ALT was 1.16 (95% CI 0.73–1.85) times more common in combined therapy over monotherapy; RR in Dummer 1.45 (95% CI 0.76–2.77), Robert 0.78 (95% CI 0.55–1.11), and Ascierto 1.48 (95% CI 1.06–2.08). Only the Ascierto trial showed statistical significance and was more common in combined therapy over monotherapy.

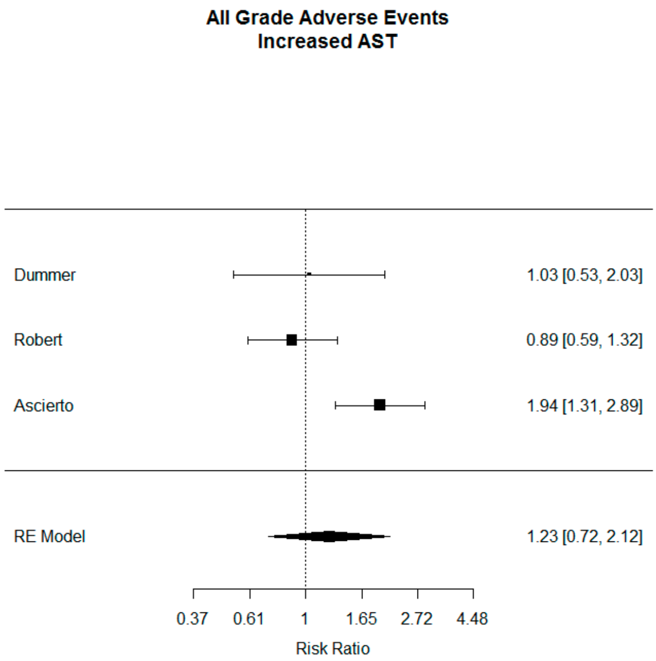

Figure S13. Relative risk (RR) of "increased AST" adverse events; all grades. Increased AST was relatively 1.23 (95% CI 0.72–2.12) times more common in combined therapy over monotherapy, with no statistical significance. Both the Dummer (RR: 1.03, 95% CI 0.53–2.03) and Robert (RR: 0.89, 95% CI 0.59–1.32) trials showed no statistical significance. In the Ascierto trial, it was relatively 1.94 (95% CI 1.31–2.89) times more common in combined therapy over monotherapy and was statistically significant.

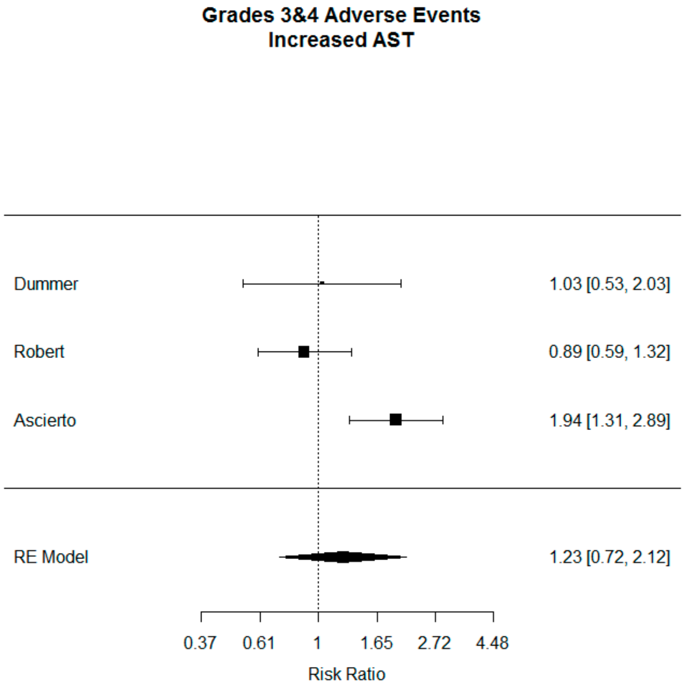

Figure 14. Relative risk (RR) of "increased AST" adverse events; grades 3 and 4. Increased AST was 1.23 (95% CI 0.72–2.12) times more common in combined therapy over monotherapy; RR in Dummer 1.03 (95% CI 0.53–2.03), Robert 0.89 (95% CI 0.59–1.32), and Ascierto 1.94 (95% CI 1.31–2.89). Only the Ascierto trial showed statistical significance and was more common in combined therapy over monotherapy.

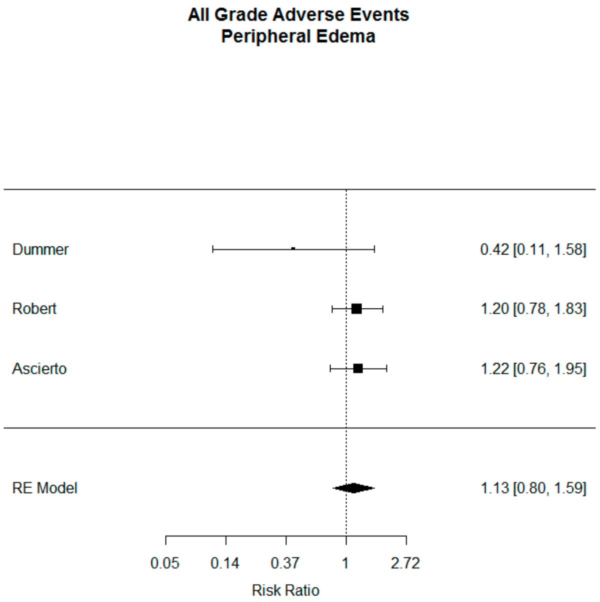

Figure S15. Relative risk (RR) of "peripheral edema" adverse events; all grades. Peripheral edema was 1.13 (95% CI 0.80–1.59) times more common in combined therapy over monotherapy. In the Dummer trial, peripheral edema was 0.42 times more common in combined therapy over monotherapy (95% CI 0.11–1.58); in the Robert trial, peripheral edema was 1.20 (95% CI 0.78–1.83) times more common in combined therapy over monotherapy; while Ascierto trail showed 1.22 (95% CI 0.76–1.95) times more common in combined therapy over monotherapy. None of the involved trials showed any statistical significance in combined therapy over monotherapy.

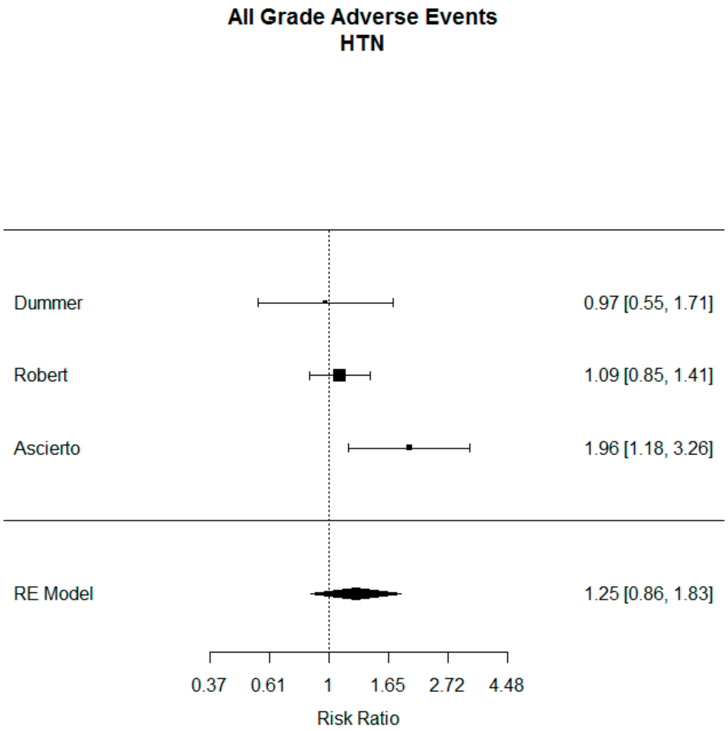

Figure S16. Relative risk (RR) of "hypertension" adverse events; all grades. Hypertension was 1.25 (95% CI 0.86–1.83) times more common in combined therapy over monotherapy. In the Dummer trial, it was 0.97 (95% CI 0.55–1.71) times more common in combined therapy over monotherapy; the Robert trial shows it to be 1.09 (95% CI 0.85–1.41) times more common in combined therapy over monotherapy; and the Ascierto trial showed combined, it was 1.96 (95% CI 1.18–3.26) times more common in combined therapy over monotherapy. Only the Ascierto trial showed statistical significance.

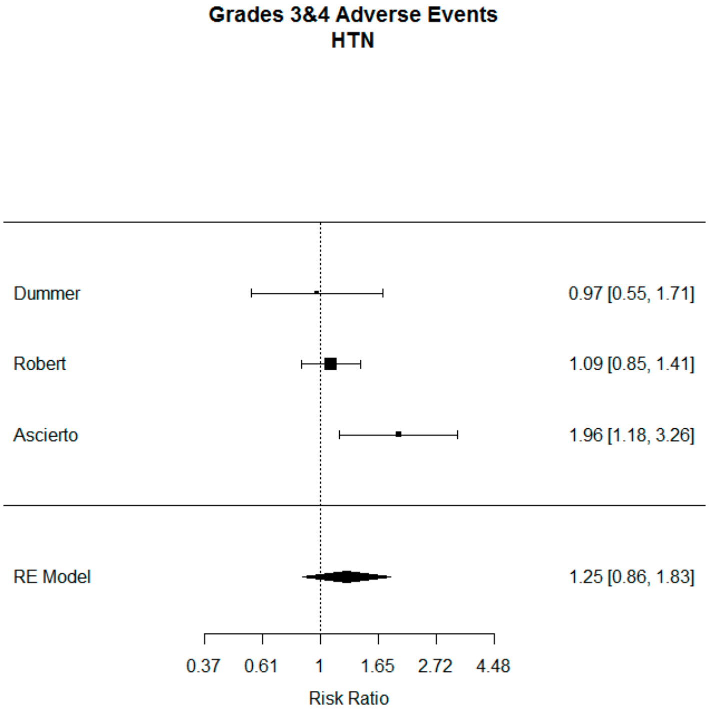

Figure S17. Relative risk (RR) of "hypertension" adverse events; grades 3 and 4. Hypertension was 1.25 (95% CI 0.86–1.83) times more common in combined therapy over monotherapy; RR in Dummer 0.97 (95% CI 0.55–1.71), Robert 1.09 (95% CI 0.85–1.41), and Ascierto 1.96 (95% CI 1.18–3.26). Only the Ascierto trial showed statistical significance and was more common in monotherapy.

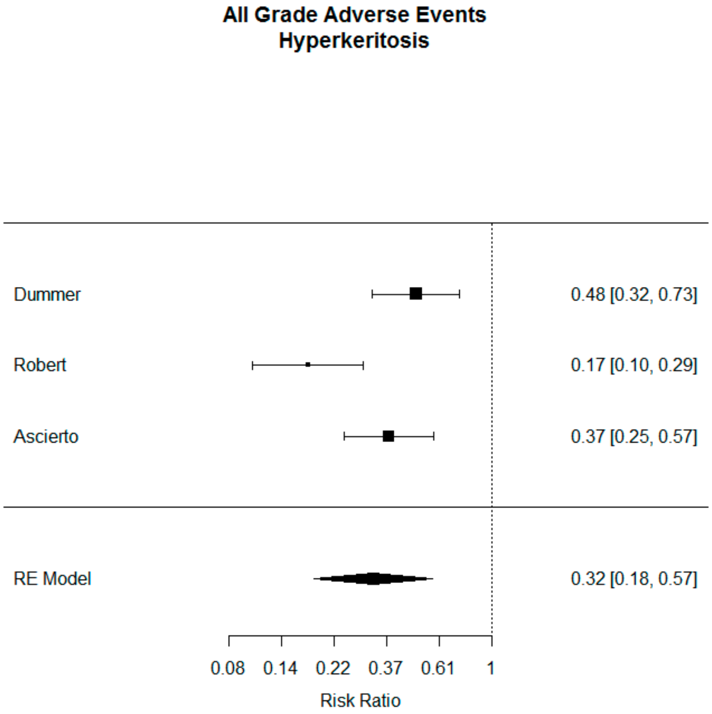

Figure S18. Relative risk (RR) of "hyperkeritosis" adverse events; all grades. There was a 0.32 (95% CI 0.18–0.57) risk in combination therapy over monotherapy for hyperkeratosis to occur. All trials showed statistical significance. RR in Dummer was 0.48 (95% CI 0.32–0.73), Robert 0.17 (95% CI 0.10–0.29), and 0.37 in the Ascierto trial (0.25–0.57) in combined therapy over monotherapy. It can also be said that encorafenib plus binimetinib combination therapy had a higher risk compared to dabrafenib plus trametinib combination therapy.

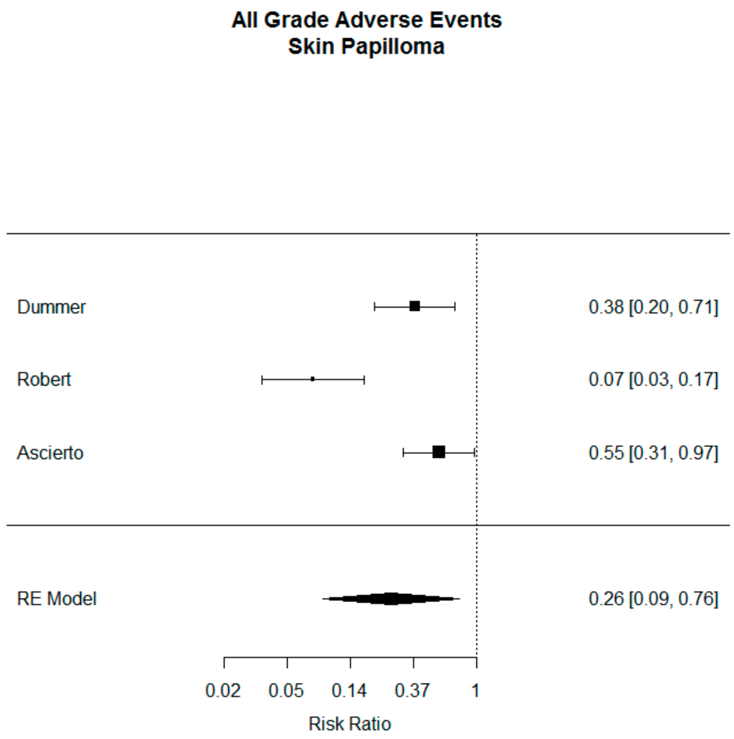

Figure S19. Relative risk (RR) of "skin papilloma" adverse events; all grades. There was a 0.26 (95% CI 0.09–0.76) risk in combination therapy over monotherapy for skin papilloma to occur. All studies showed statistical significance. RR in Dummer was 0.38 (95% CI 0.20–0.71), Robert 0.07 (95% CI 0.03–0.17), and Ascierto 0.55 (95% CI 0.31–0.97) in combined therapy over monotherapy. It can also be said that dabrafenib plus trametinib combination therapy had a lower risk compared to vemurafenib plus cobimetinib and encorafenib plus binimetinib combination therapies.

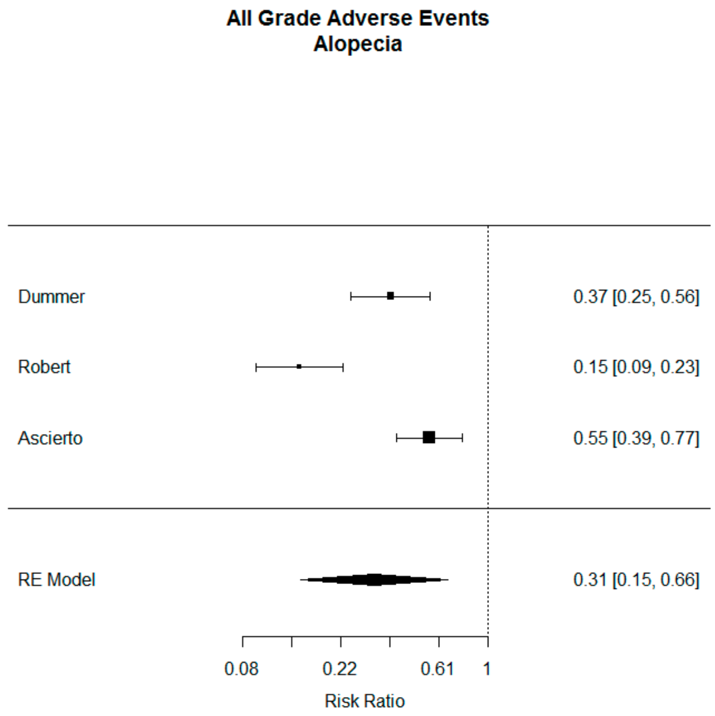

Figure S20. Relative risk (RR) of "alopecia" adverse events; all grades. There was a 0.31 (95% CI 0.15–0.66) risk in combination therapy over monotherapy for alopecia to occur. All trials showed statistical significance. RR in Dummer was 0.37 (95% CI 0.25–0.56), Robert 0.15 (95% CI 0.09–0.23), and Ascierto 0.55 (95% CI 0.39–0.77) in combined therapy over monotherapy. It can also be said that dabrafenib plus trametinib combination therapy had a lower risk compared to vemurafenib plus cobimetinib. and encorafenib plus binimetinib combination therapies.

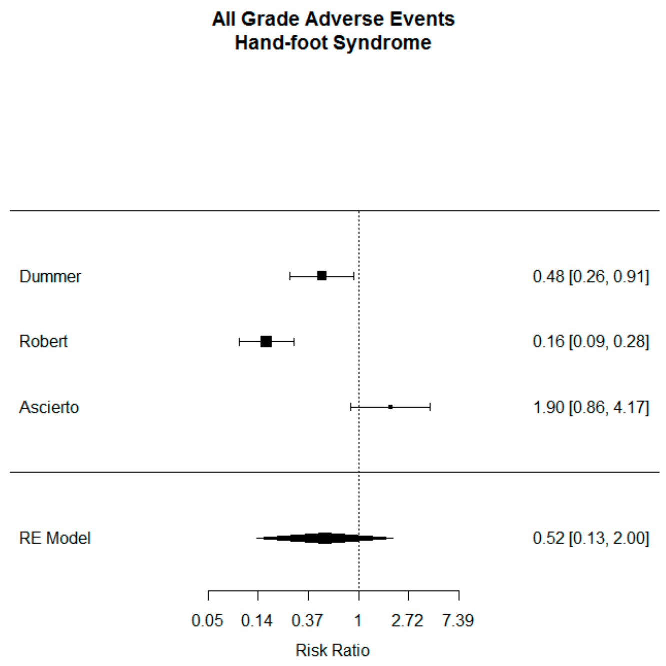

Figure S21. Relative risk (RR) of "hand-foot syndrome" adverse events; all grades. There was a 0.52 (95% CI 0.13–2.00) risk in combination therapy over monotherapy for hand-foot syndrome to occur. RR in Dummer was 0.48 (95% CI 0.09–0.28) and Robert 0.16 (95% CI 0.09–0.28) in combined therapy over monotherapy. Both of the above studies showed statistical significance. Relative risk in Ascierto was 1.90 (95% CI 0.86–4.17) in combined therapy over monotherapy and showed no statistical significance. It can also be said that dabrafenib plus trametinib combination therapy had a lower risk compared to vemurafenib plus cobimetinib.

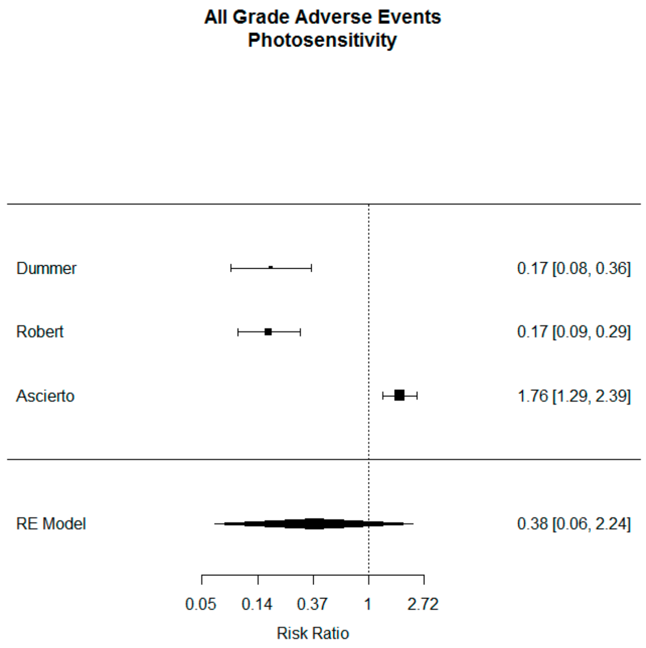

Figure S22. Relative risk (RR) of "photosensitivity" adverse events; all grades. There was a 0.38 (95% CI 0.06–2.24) relative risk in combination therapy over monotherapy for photosensitivity to occur. The Dummer (RR: 0.17, 95% CI 0.08–0.36) and Robert (RR: 0.17, 95% CI 0.09–0.29) trials showed that photosensitivity was more common in monotherapy, but the Ascierto trial showed photosensitivity (RR: 1.76, 95% CI 1.29–2.39) was more common in combined therapy over monotherapy. It can also be said that encorafenib plus binimetinib and dabrafenib plus trametinib combination therapies had a lower risk compared to vemurafenib plus cobimetinib.
